# Impact of soy- and milk-based yogurts on enamel preservation: erosion and abrasion under simulated conditions

**DOI:** 10.1007/s00784-026-06952-2

**Published:** 2026-06-04

**Authors:** Rafaela Ricci Kim, Aline Silva Braga, Gabriela Pellizon Floret, Ariadne Roehler, Jacob Schultheiss, Ana Carolina Magalhães, Marcella Esteves-Oliveira

**Affiliations:** 1 Department of Biological Sciences, Bauru School of Dentistry - University of São Paulo, Al. Octávio Pinheiro Brisolla, 9-75, Bauru, SP 17012-901 Brazil; 2 Department of Medical Materials Science and Technology, Institute for Biomedical Engineering, University Hospital Tübingen, Tübingen, Germany; 3 Department of Conservative Dentistry, Periodontology and Endodontology, Faculty of Medicine, University of Tübingen, Tübingen, Germany

**Keywords:** Abrasion, Enamel erosion, Erosive tooth wear, Fluorides, Soy-based yogurt

## Abstract

**Objectives:**

This study evaluated the effect of soy-based and milk-based yogurts against enamel erosive-abrasive surface loss.

**Materials and methods:**

Ninety-eight bovine enamel specimens were randomly allocated to seven experimental groups (*n* = 14). Over seven days, specimens underwent six daily erosive cycles (0.05 M citric acid, pH 2.3, 2 min), interspersed with remineralizing solution (pH 6.5). Post-acid preventive measures consisted of immersion in milk-based yogurt (pH 4.4), soy-based yogurt (pH 4.7), or tap water for 3 min twice daily. Under erosion-abrasion conditions, specimens underwent simulated toothbrushing using stannous ions-containing fluoride toothpaste. Fluoride-free toothpaste served as abrasion control. Enamel surface loss was measured after 7 days using three-dimensional laser scanning microscopy. Calcium and phosphorus contents were quantified by optical emission spectrometry.

**Results:**

Abrasion with a stannous ions-containing fluoride toothpaste combined with soy-based yogurt resulted in significantly lower enamel surface loss (7.9±3.3 μm) compared with the fluoride toothpaste only (12.2 ± 2.7 μm; *p*<0.05). Under erosion-only conditions, no significant differences were detected among preventive measures. Milk-based yogurt presented significantly higher calcium and phosphorus contents (Ca: 1434.1±190.3 µg/g; P: 1033.8±128.1 µg/g) than soy-based yogurt (Ca: 168.4±15.5 µg/g; P: 623.2±47.7 µg/g).

**Conclusions:**

The tested soy yogurt may offer adjunctive protection in patients exposed to erosive and abrasive challenges.

**Clinical relevance:**

The tested soy yogurt significantly enhances anti-erosive efficacy of fluoride-stannous formulations, providing clinicians with an evidence-based dietary intervention to optimize preventive strategies against erosive enamel loss.

## Introduction

Erosive tooth wear (ETW) is defined as the progressive and irreversible loss of dental hard tissues, mainly enamel and dentin, caused by the chemical action of acids without bacterial involvement. This process may be exacerbated by associated mechanical factors, such as abrasion, commonly related to toothbrushing, or attrition [1–3]. ETW is a clinical and conceptual term that describes the cumulative outcome of chemical erosion, often modified by mechanical processes. In experimental studies, this condition is commonly quantified as enamel surface loss or wear, which refers to the measurable irreversible loss of mineralized enamel tissue over time, typically expressed in micrometer [4].

The acids affecting dental structures may have an extrinsic origin, derived from dietary sources (soft drinks, citrus fruits, vinegars, among others), or an intrinsic origin, resulting from conditions such as gastroesophageal reflux or frequent vomiting [5, 6]. Epidemiological studies have reported a high prevalence of this condition, estimated to range from 30% to 50% in primary dentition and from 20% to 45% in permanent dentition [7, 8].

The progression of ETW may significantly impair patients’ quality of life, being associated with dentin hypersensitivity, functional impairments such as chewing difficulties, and esthetic concerns, including tooth discoloration and shortening, which may negatively affect self-esteem and smile appearance [9]. Given its multifactorial nature, the clinical management of ETW requires careful assessment of the chemical, biological, and behavioral factors involved, as well as the implementation of evidence-based preventive strategies [10–12].

Among the natural protective factors against erosion, saliva plays a fundamental role by neutralizing and clearing acids, promoting the formation of the acquired pellicle, and providing calcium and phosphate ions necessary for remineralization processes [13]. Similarly, certain foods, particularly dairy products such as milk and cheese, have been associated with protective effects on enamel, mainly attributed to their mineral, protein and lipidic content, in especial to calcium interactions with the dental surface [14–16]. Previous studies have demonstrated that, although acidic foods increase dental erosion, the consumption of milk and milk-based yogurt may attenuate their effect [16, 17].

Yogurt presents properties comparable to those of milk due to the presence of calcium, phosphate, and proteins, especially casein at high concentration. These components may contribute to enamel remineralization and to the formation of a protective layer on the tooth surface [16–19]. Evidence indicates that casein and its derivatives, such as casein phosphopeptides (CPPs), can adsorb onto enamel, forming a physical barrier against acids and promoting the retention of calcium and phosphate ions, thereby reducing demineralization [20, 21].

Concurrently, there has been a markable increase in the consumption of plant-based foods (vegetarian and vegan style of life), driven by changes in dietary habits and growing environmental and health concerns [22–26]. In this context, plant-based yogurts have gained attention as alternatives to traditional milk-yogurt products.

Recent studies have indicated that plant-based yogurts may exhibit a protective effect on dental enamel, even in the absence of milk-derived proteins such as casein [27, 28]. This protective potential has been suggested to be related to the physicochemical characteristics of these products, including mineral content, buffering capacity, and the food matrix, rather than exclusively to the presence of casein-derived peptides. These findings highlight the need to further evaluate the role of plant-based yogurt formulations in their interaction with dental hard tissues, particularly considering their increasing consumption [27–29]. The available evidence remains limited, particularly regarding the mechanisms involved and the interaction of these products with other preventive strategies commonly adopted in clinical practice, such as the use of fluoridated toothpastes. To the best of our knowledge, no study has evaluated the protective effect of soy-based yogurt in an erosion–abrasion model or its interaction with fluoridated toothpastes.

Moreover, many previous studies have employed experimental models based on continuous acidic exposure or prolonged contact times with foods or beverages, which do not adequately reflect the clinical dynamics of oral cavity [30–33]. Clinically, erosive challenges are typically short and repeated, interspersed with periods of salivary recovery and often associated with toothbrushing abrasion. Consequently, it remains unclear whether the protective effects attributed to foods, such as yogurt, persist under more clinically relevant conditions, especially when erosion is combined with abrasion.

Therefore, the present study aimed to evaluate the protective effects of milk-based and soy-based yogurts on enamel surface loss under erosive conditions alone and when combined with abrasion, with particular emphasis on their interaction with the use of a stannous ions-containing fluoride toothpaste following erosive challenges.

## Materials and methods

### Experimental design

**Fig. 1:**
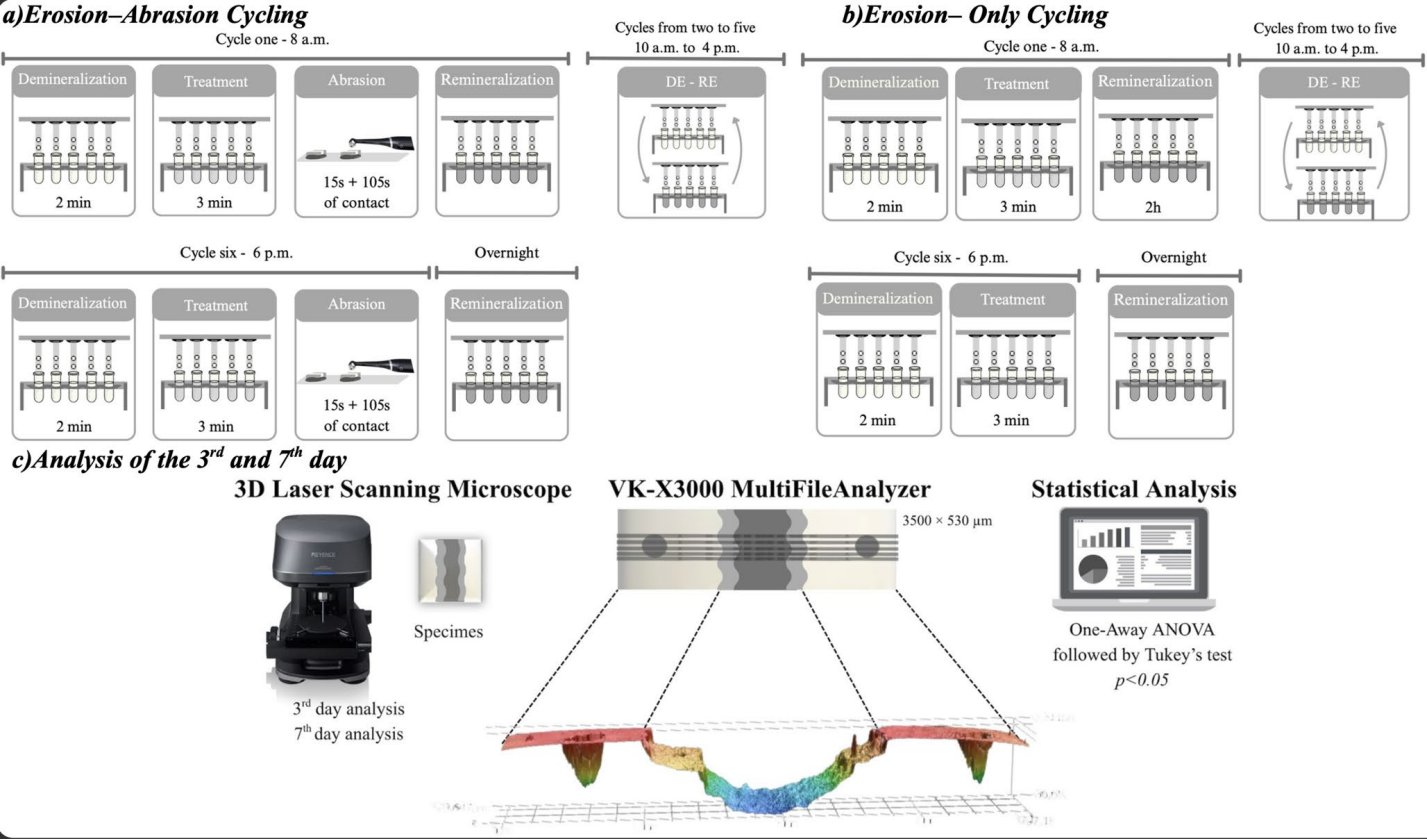
Experimental design and analytical workflow

The experimental protocol was designed to simulate clinical preventive recommendations for erosive tooth wear, namely the consumption of yogurt immediately after acidic food or beverage intake, followed by toothbrushing with a stannous ions-containing fluoride toothpaste. This in vitro model enabled the systematic evaluation of post-acid exposure preventive measures under controlled erosive–abrasive cycling conditions. The experimental design is illustrated in Fig. 1a.

## Sample size calculation

A sample size of 14 specimens per group was determined based on data from Esteves-Oliveira et al. [34] In that study, the difference in enamel surface loss between the control group (27.22±4.1 μm) and the stannous ions-containing fluoride group (3.31±2.0 μm) was 23.91 μm, with a pooled standard deviation of 3.23 μm, resulting in a standardized effect size (Cohen’s d) of approximately 7.41. Assuming an alpha level of 0.05 and a target power of 0.80, this contrast would require fewer than one specimen per group. Therefore, the allocation of 14 specimens per group provides statistical power exceeding 99% for this benchmark effect.

## Tooth sample preparation

A total of 98 enamel specimens were prepared from permanent bovine incisors and sectioned into dimensions of 4 mm⋅4 mm using two diamond discs mounted on a IsoMet^®^ Low Speed Precision Cutter (Buehler^®^, Lake Bluff, USA).Throughout the study, specimens were stored in a 0.1% thymol solution, and only specimens without visible cracks or structural defects were included.

Enamel surfaces were planarized and polished under continuous water irrigation using a Micro Grinder 400 polishing machine (Exakt^®^, Norderstedt, Germany).Each specimen was fixed with sticky wax at the center of an acrylic plate and initially polished from the dentin side, followed by the enamel side, to obtain planar and parallel surfaces. Final enamel polishing was performed sequentially using 800, 1200, 2400, and 4000 grit silicon carbide papers under water cooling, followed by a 2 min polishing step with a felt disc and diamond suspension.Enamel reduction by polishing was monitored using a micrometer standardized not to exceed 400 μm [34].

The absence of dentin exposure was confirmed using a 3D laser scanning microscope (VK-3000, Keyence^®^, Osaka, Japan). Specimens were subsequently cleaned in distilled water using an ultrasonic bath and stored in 0.1% thymol at 4 °C until use [34].

To ensure that enamel surface loss was measured consistently at the same location throughout the experiment, a procedure was adopted to guarantee positional accuracy, as previously described in studies by our group [34]. For this purpose, two dots were made in the control area of each sample using a spherical bur (No. 1011) (Hager & Meisinger^®^, Neuss, Germany), one to the left and one to the right of the experimental area. The control areas of each specimen were protected with removable adhesive tape during the erosive-abrasive cycling and removed only during the analysis [34].

## Experimental groups

Specimens were allocated into seven experimental groups to isolate the effects of yogurt type (milk-based vs. soy-based) and brushing abrasion (Table 1). The inclusion of a fluoride-free toothpaste group served to control for the well-established protective effect of stannous ions-containing fluoride toothpastes against abrasion of eroded enamel surfaces.

All experimental groups were subjected to the same erosive and remineralization protocol. The experimental variables were the presence or absence of brushing abrasion, the use of fluoride-containing or fluoride-free toothpaste (Table 1), and the type of post-acid treatment (milk-based yogurt, soy-based yogurt or water), as summarized in Table 2.

**Table 1:** Composition of yogurt products and toothpastes

Product type	Commercial name	Composition
Soy-based yogurt	Rewe Vegan Soja Natur	Water, soy (13.3%), modified starch, starch, sea salt, natural flavoring, yogurt cultures
Milk-based yogurt	Rewe Bio Yogurt Mild 3.8% fat	Milk and yogurt cultures
Toothpaste type		**Full composition**
Tin-containing fluoride toothpaste Elmex^®^ Opti-namel (Colgate-Palmolive^®^, Świdnica, Poland)		Aqua, glycerin, sorbitol, hydrated silica, hydroxyethylcellulose, aroma, cocamidopropyl betaine, titanium dioxide, olaflur (AmF), sodium gluconate, stannous chloride, alumina, chitosan, sodium saccharin, sodium fluoride, potassium hydroxide, hydrochloric acid
Fluoride-free toothpaste Nenedent^®^ children’s (Dentinox^®^, Berlin, Germany)		Aqua, hydrated silica, glycerin, xylitol, propylene glycol, xanthan gum, aroma, sodium lauroyl sarcosinate, disodium EDTA, sodium chloride, sodium hydroxide

**Table 2:** Experimental groups and preventive conditions

Group	Post-Acid Treatment	Abrasion	Toothpaste (brand)	pH
SY + Sn/F	Soy-based	Yes	Elmex^®^ Opti-namel	4.75
MY + Sn/F	Milk-based	Yes	Elmex^®^ Opti-namel	4.75
W + Sn/F	Water	Yes	Elmex^®^ Opti-namel	4.75
W + NF	Water	Yes	Nenedent^®^ Children’s	7.89
MY	Milk-based	No	–	–
SY	Soy-based	No	–	–
W	Water	No	–	–

## Erosive and abrasive cycling

Custom-made racks with two rows of Falcon tubes allowed precise and simultaneous handling of specimen groups across all stages of the erosion–abrasion cycle (Fig. 1a). Demineralizing and remineralizing solutions were placed in tubes containing 45 mL each, whereas treatment solutions (milk-based yogurt, soy-based yogurt, or tap water) were placed in tubes containing a volume of 25 mL. All solutions were maintained at room temperature (25 °C) and were changed before each exposition.

Six erosive cycles per day were performed for seven consecutive days, at 8:00 a.m., 10:00 a.m., 12:00 p.m., 2:00 p.m., 4:00 p.m., and 6:00 p.m. Each erosive cycle consisted of immersion in 0.05 M citric acid (pH 2.3, 25 °C) for 2 min, followed by rinsing with deionized water for 5s. After each erosive cycle, specimens were stored in a remineralizing solution.

At 8:00 a.m. and 6:00 p.m., immediately after the first and last erosive challenges of the day, the treatment groups (soy-based yogurt, milk-based yogurt or water, both with or without brushing) were immersed in their respective treatment solutions for 3 min and rinsed with deionized water.

For brushing groups, immediately after the treatment, the procedure was performed for 15 s using an electric toothbrush (Braun Oral-B Professional Care 8500, Precision Clean brush head) operating at approximately 8.800 oscillations and 40.000 pulsations per minute, mounted on a custom-designed 3D-printed brushing device, ensuring a constant load of 1.5 N and a standardized brushing motion for all specimens [35]. Immediately after brushing, specimens were immersed for 105 s in a toothpaste slurry (1 part toothpaste to 3 parts deionized water), resulting in a total toothpaste contact time of 2 min. Specimens were then rinsed with deionized water and stored in the remineralizing solution.

Between erosive challenges throughout the day, specimens were maintained for 2 h in a supersaturated remineralizing solution containing 4.08 mM H₃PO₄, 20.10 mM KCl, 11.90 mM Na₂CO₃, and 1.98 mM CaCl₂ (pH 6.5), as previously described by Gerrard and Winter [36]. The remaining four daily challenges (10:00 a.m., 12:00 p.m., 2:00 p.m., and 4:00 p.m.) consisted exclusively of immersion in the citric acid solution for 2 min, followed by rinsing and storage in the remineralizing solution.

The three experimental groups without abrasion (soy-based yogurt, milk-based yogurt and water) were subjected exclusively to the erosive-cycling but did not receive brushing or toothpaste slurry application. These groups were therefore exposed exclusively to the erosive challenge, followed by the corresponding post‑acid treatments (twice a day) and remineralization phases (Fig. 1b).

After completion of the six daily erosive cycles, all specimens, regardless of experimental group, were stored overnight in the remineralizing solution, simulating an extended period without acidic challenges.

### Scanning of enamel surfaces

Enamel surface profiles were evaluated after the third (day 3) and seventh (day 7) day of the erosive cycling protocol. After day 3, all specimens were rinsed with deionized water, and the adhesive tapes protecting the control areas were carefully removed. Each specimen presented a central experimental area exposed to the erosive challenges, laterally delimited by two control areas marked on the right and left sides.

Surface topography was analyzed using a 3D laser scanning microscope (VK-X3000, Keyence^®^, Osaka, Japan). The analyzed area comprised the central experimental region and the two adjacent control areas, resulting in a standardized measurement area of 3500⋅530 μm. Scans were performed using a 20⋅ objective lens, as previously described [37].

After scanning on day 3, the adhesive tapes were repositioned in their original locations, and specimens returned to the erosive cycling protocol until day 7. At the end of day 7, the same cleaning and scanning procedures were repeated.

## Analysis of enamel surface loss

Image analysis was performed using the VK-X3000 MultiFileAnalyzer software (version 3.3.1.85, Keyence^®^,Osaka, Japan). The software processes the full 3D height map of the scanned area, containing millions of height points. Surface leveling was performed by selecting two reference regions within the control areas, from which the software generated a leveled reference plane.

A representative mean height profile was then computed by averaging the height values across the entire width of the scan. Enamel surface loss was quantified as the vertical distance between the leveled reference plane and the mean eroded surface, expressed in micrometers (µm). To ensure precision the used tool performed the same measurements using 25 parallel line scans spaced at 30 μm intervals across the experimental and control areas (Fig. 1c).

## Analysis of calcium and phosphorus content: inductively coupled plasma optical emission spectrometry (ICP-OES) and pH

Milk-based and soy-based yogurt samples, as well as a blank control, were analyzed in six replicates per group (*n*=6). All samples were subjected to the same acid digestion procedure. Briefly, 1.00±0.01 g of each yogurt sample was placed in a borosilicate glass tube and treated with 5 mL of 65% HNO_3_ and 1 mL of 30% H_2_O_2_. A blank tube containing the digestion reagents, but no yogurt sample was prepared as a control [38].

The tubes were covered with borosilicate watch glasses and heated at 80 °C for 24 h to ensure complete digestion. Digestion vessels were weighed before and after heating to assess potential mass loss during the digestion process. After digestion, the samples were diluted 1:10 (v/v) with 2% HNO_3_.

Elemental analysis of calcium (Ca) and phosphorus (P) was performed using an inductively coupled plasma optical emission spectrometer Avio^®^220 Max (PerkinElmer^®^, Waltham, USA), following standard procedures. Yttrium (20 µg/mL) was used as an internal standard [39].

Measured concentrations were corrected by subtracting the values obtained from the blank control group, followed by multiplication by the dilution factor and adjustment based on recovery of the internal standard.

The pH of yogurts were quantified using a 691 pH meter (Metrohm^®^, Filderstadt, Germany) under continuous stirring to prevent ion accumulation and ensure stable readings; the electrode was immersed directly in the original retail container, and measurements were recorded after stabilization.

### Statistical analysis

Statistical analysis was performed using GraphPad Prism (GraphPad Software^®^, San Diego, USA). The data passed the normality and homogeninity tests. Thereafter, enamel surface loss data were analyzed using one-way analysis of variance (ANOVA), with treatment as fixed factor, followed by Tukey’s post hoc test for multiple comparisons. Calcium and phosphorus concentrations were compared using an unpaired *t*-test with Welch’s correction to account for potential heterogeneity of variances. All tests were two-tailed, and the significance level was set at α= 0.05.

## Results

### Erosion–abrasion cycling

**Fig. 2:**
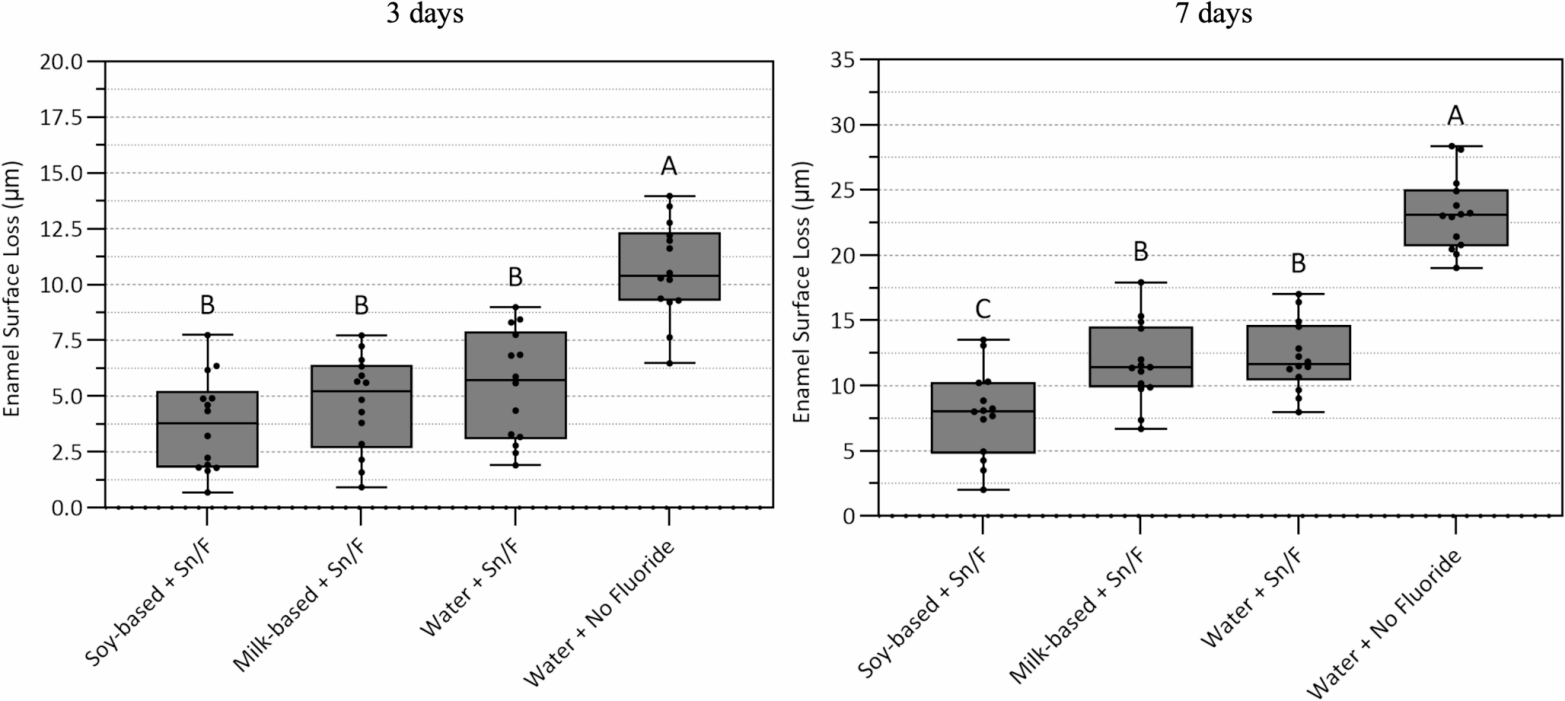
Erosion plus abrasion. Mean enamel surface loss (µm) after 3 and 7 days. Data were analyzed using one-way ANOVA followed by Tukey’s post hoc test (factors: treatment, for 3 days *p* < 0.0001 and for 7 days *p = < 0.0001*; *n* = 14). Different uppercase letters indicate statistically significant differences among treatment groups

Enamel surface loss under erosive–abrasive conditions are shown in Fig. 2. At day 3, the water+no fluoride (negative control) group exhibited the highest mean enamel surface loss, differing significantly from the milk-based yogurt + Sn/F, soy-based yogurt+Sn/F, and water + Sn/F (*p<0.0001*, Fig. 2).

By day 7, enamel surface loss increased in all experimental groups (Fig. 2). The water + no fluoride group continued to show the greatest enamel loss values and differed significantly from all other groups (*p<0.0001*). The soy-based yogurt+Sn/F group showed the lowest enamel surface loss at day 7, differing significantly from the milk-based yogurt+Sn/F, water+Sn/F, and water+no fluoride groups (Fig. 2).

### Erosion–only cycling

**Fig. 3:**
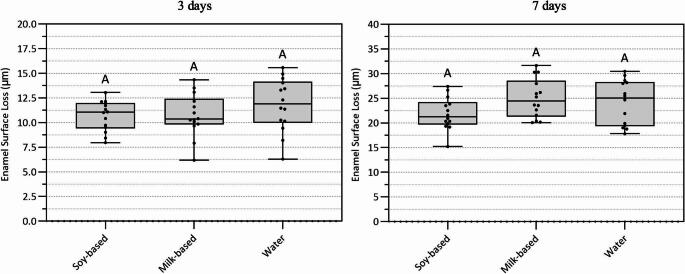
Erosion Only. Mean enamel surface loss (µm) after 3 and 7 days under erosive conditions without abrasion. Data were analyzed using one-way ANOVA followed by followed by Tukey’s post hoc test (factors: treatment, for 3 days *p =* 0.3236 and for 7 days *p* = 0.1091; *n* = 14). Different uppercase letters indicate statistically significant differences among treatment groups

Enamel surface loss under erosive conditions without abrasion is presented in Fig. 3. At both day 3 and day 7, comparison among treatments (soy-based yogurt, milk-based yogurt, and water) revealed no statistically significant differences in enamel surface loss, as indicated by identical uppercase letters (*p = 0*,*3236* and *p* = 1091 respectively, Fig. 3).

### Analysis of pH, calcium, and phosphate

The pH, calcium, and phosphorus values of the yogurt specimens are shown in Table 3. The milk-based yogurt presented a lower pH (4.4) than the soy-based yogurt (4.7). Calcium concentration was significantly higher in the milk-based yogurt (1434.1±190.3 µg/g) compared with the soy-based yogurt (168.4±15.5 µg/g; *p*<0.0001). Similarly, phosphorus concentration was higher in the milk-based yogurt (1033.8±128.1 µg/g) than in the soy-based yogurt (623.2±47.7 µg/g; *p*=0.0004).

**Table 3:** pH and mineral composition (calcium and phosphorus) of the yogurts

Yogurt	pH	Calcium (µg/g)	Phosphorus (µg/g)
Soy-based	4.70	168.48 ± 15.46	623.18 ± 47.73
Milk-based	4.36	1434.10 ± 190.31	1033.80 ± 128.09

The mineral composition (calcium and phosphorus) of the milk-based and soy-based yogurts, determined by inductively coupled plasma optical emission spectrometry (ICP-OES). Data are presented as mean ± standard deviation (*n* = 6). Unpaired t test with Welch´s correlation, Calcium *p* < 0.0001 and Phosphorous *p* = 0.0004

## Discussion

The results of the present study demonstrated that the combination of soy-based yogurt with a toothpaste containing fluoride and stannous ions (1450 ppm F, 3500 ppm Sn, 0.5% chitosan).The selection of Elmex^®^ Opti-namel toothpaste (formerly known as Elmex^®^ Erosion Protection) is based on its long-standing recognition in the scientific literature, having served as a -gold-standard toothpaste applied to prevent erosive tooth wear for approximately 12 years [40–43], under erosion–abrasion conditions, resulted in the lowest enamel loss values. The same protective effect was not observed when the soy-based yogurt was applied only between erosive challenges, in the absence of fluoride or stannous ions, indicating that components of this yogurt likely react with residual components of the fluoridated toothpaste, both present on the dental surface, thereby protecting the enamel against erosive loss.

It is important to emphasize that, in the present study, the effect of both the soy-based yogurt and the milk-based yogurt was observed only when combined with a toothpaste containing fluoride and stannous ions. The literature consistently demonstrates that fluoridated toothpastes, especially when associated with stannous compounds, significantly reduce enamel loss under in in vitro erosion–abrasion models [44, 45].

The combination of fluoride and stannous ions is considered an effective and safe agent, presenting superior benefits compared with other fluoridated compounds, in addition to positive effects on dental plaque, gingiva, and dentin hypersensitivity [45–48].

Fluoride and stannous ions interact directly with hydroxyapatite, the main mineral component of dental enamel. Stannous chloride (SnCl₂), being soluble in aqueous media, releases Sn²⁺ ions, which exhibit high affinity for the phosphate groups of hydroxyapatites, forming a modified mineral layer enriched with stannous ions and fluoride that offers mechanical protection. The formation of this layer reduces the solubility of calcium phosphate under acidic conditions and decreases calcium release during erosive challenges, thereby increasing resistance to enamel loss [47].

This is the first study to evaluate a soy-based yogurt regarding erosive enamel loss, and despite its protective effect against enamel loss when combined with a fluoridated toothpaste, its calcium and phosphate contents were significantly lower than those of the milk-based yogurt, which showed a comparatively lower protective effect in this study. Therefore, the mechanism of action of this soy-based yogurt cannot be explained by its calcium and phosphate content.

The starch present in the formulations of vegan yogurts plays a fundamental role in the structure and stability of these products, compensating for the absence of casein, which is the main protein responsible for the firm texture of traditional dairy yogurts. Its function as a thickening agent provides greater body and creaminess, ensuring a less liquid consistency. In addition, it acts as a stabilizer, preventing phase separation, reducing syneresis, and maintaining viscosity during storage. From a sensory perspective, starch improves the mouthfeel, providing softness and density like those of traditional yogurt. Technically, it partially replaces the function of animal proteins by forming a network that retains water and supports the structure of the product [49]. Another relevant aspect may be its ability to attenuate perceived acidity: the increase in viscosity can limit the diffusion of hydrogen ions in the mouth and modify oral exposure dynamics, which could reduce the sensory perception of sharp acidity. This effect might also influence the erosive potential compared with low-viscosity, strongly acidic beverages (e.g., citric acid–based drinks), but this hypothesis requires targeted investigation.

The presence of starch and modified starch in the soy-based yogurt may have contributed to the increased viscosity of the surface layer, favoring longer contact time with enamel surface and more stable mineral layer enriched with stannous ions and fluoride, a factor recognized as relevant for modulation of tooth erosion [21, 32]. More viscous media tend to reduce clearance and the immediate removal of active substances from the dental surface, potentially delaying the diffusion and elimination of fluoride ions and stannous ions species, whose efficacy depends on surface retention and interaction with the enamel–medium interface [33, 39]. Thus, it is plausible that the starch-rich matrix of the soy-based yogurt enhanced the action of the toothpaste containing fluoride, stannous ions, and chitosan, although this mechanism still requires direct experimental confirmation [22, 46].

In the literature, the study by Dos Santos et al. [50] subjected enamel specimens to a static continuous erosion model, with direct immersion in beverages for 120 min without remineralization phases. The soy-based beverages were commercially available products (Addes^®^, Unilever) with pH varying from 3.9 to 4.2, while the non–soy-based beverages comprised pH values from 2.9 to 3.4. Under these conditions, beverages containing soy extract promoted lower enamel surface loss and smaller increases in surface roughness compared with non-soy beverages, although all drinks exhibited erosive potential. This attenuating effect of soy should be interpreted with caution, as prolonged and continuous acidic exposure favors chemical interactions between beverage components and the enamel surface and does not reflect the clinical dynamics of short acidic challenges interspersed with periods of salivary recovery. Furthermore, they tested beverage and not yogurt, which are more acidic and less viscous.

In the case of fermented dairy products, even milk yogurt with pH around 4.4, its calcium and phosphorus content plays an important protective role, maintaining the food saturated with respect to hydroxyapatite, reducing or avoiding the chemical gradient required for mineral dissolution. Instead, this type of food is known to have remineralizing potential [51, 52]. With this respect, past in vitro erosive protocols frequently involved prolonged and repeated acidic exposures with accumulative several hours of contact time with milk-derived foods either [29, 53], conditions that are far from clinical reality. In these aggressive experimental models, the prior application of milk and yogurt and extended storage in artificial saliva may have artificially enhanced the apparent reduction in demineralization. In addition, the use of outcome measures such as dental weight loss [53] or even confocal microscopy [29] limits clinical interpretation. Similarly, sequential demineralization–remineralization models, in that eroded enamel is immersed in milk or artificial saliva for up to 3 h a prolonged period of exposure even with the application of sensitive methods to detect surface and mechanical changes, does not reflect real oral exposure patterns, restricting direct clinical extrapolation neither [54].

Therefore, the past protective effects attributed to yogurt should be interpreted cautiously, especially in continuous demineralization models in which dairy components remain present during acidic challenge, potentially overestimating protection [23]. In contrast, pH-cycling models that simulate short and repeated acidic challenges with remineralization periods better approximate clinical conditions. Under these circumstances, a previous study showed that fluoridated milk had greater protective potential, whereas non-fluoridated dairy products demonstrated limited effects gainst erosive enamel loss [16] consistent with the findings of the present study.

Overall, evidence suggests that many of the past experimental protocols overestimate the protective effects of milk and yogurt, due to prolonged chemical interaction with enamel. Models incorporating short, repeated erosive challenges, salivary recovery, and abrasion when applicable, consistently demonstrate more limited protective effects, reinforcing that experimental design has a decisive influence on the magnitude and direction of the reported outcomes [16, 23, 29, 50].Therefore, the protective effect of milk and derivates may be of less relevance in case of ETW under clinical conditions, which needs to be further investigated.

Finally, limitations inherent to the in vitro model should be considered, such as the absence of human saliva, acquired pellicle formation, and physiological remineralization mechanisms, which favor progression of erosive loss at a higher rate than would occur in vivo [32]. On the other hand, inclusion of the erosion-only condition allowed isolation of the effect of yogurts on enamel, eliminating the influence of mechanical factors and fluoridated toothpaste, thereby contributing to a better understanding of the mechanisms involved.

## Conclusion

Within the limitations of this in vitro erosion–abrasion study, the combination of the tested soy yogurt with a fluoride–stannous toothpaste significantly reduced enamel surface loss, whereas the milk-based yogurt did not. The enhanced protection does not appear to be related to the yogurt’s mineral content, but may be associated with matrix-related factors that favor interaction with residual fluoride–stannous deposits on enamel, although this mechanism requires confirmation. Further in situ and clinical studies are required to confirm the clinical significance of these findings.

Overall, these findings emphasize that protection against erosive enamel loss under clinically relevant cycling conditions is primarily attributable to fluoride, particularly when combined with stannous compounds. Yogurt, soy or milk-based, alone provides limited benefit.

## Data Availability

All data generated or analyzed during this study are included in this article and its supplementary material files. Further inquiries can be directed to the corresponding authors.

## References

[1] Carvalho TS, Lussi A (2014) Combined effect of a fluoride-, stannous- and chitosan-containing toothpaste and stannous-containing rinse on the prevention of initial enamel erosion-abrasion. J Dent 42(4):450–459. 10.1016/j.jdent.2014.01.004 24440712 10.1016/j.jdent.2014.01.004

[2] Schlueter N, Amaechi BT, Bartlett D, Buzalaf MAR, Carvalho TS, Ganss C et al (2020) Terminology of Erosive Tooth Wear: Consensus Report of a Workshop Organized by the ORCA and the Cariology Research Group of the IADR. Caries Res 54(1):2–6. 10.1159/000503308 31610535 10.1159/000503308

[3] FDI World Dental Federation (2024) Erosive tooth wear: terminology, diagnosis and risk assessment. Int Dent J 74(1):1–937479594

[4] Carvalho TS, Baumann T, Lussi A (2017) Does erosion progress differently on teeth already presenting clinical signs of erosive tooth wear than on sound teeth? An in vitro pilot trial. BMC Oral Health 17:14. 10.1186/s12903-016-0231-y 10.1186/s12903-016-0231-y PMC4948097 27430320

[5] Chan AS, Tran TTK, Hsu YH, Liu SYS, Kroon J (2020) A systematic review of dietary acids and habits on dental erosion in adolescents. Int J Paediatr Dent 30(6):713–733. 10.1111/ipd.12643 32246790 10.1111/ipd.12643

[6] Nijakowski K, Jankowski J, Gruszczyński D, Surdacka A (2023) Eating disorders and dental erosion: a systematic review. J Clin Med 12:6161. 10.3390/jcm12196161 37834805 10.3390/jcm12196161 PMC10573129

[7] Schlueter N, Luka B (2018) Epidemiology of dental erosion—a review of the literature. Caries Res 52(4):265–277. 10.1038/sj.bdj.2018.167

[8] Marschner F, Kanzow P, Wiegand A (2025) Systematic review and meta-analysis on prevalence and anamnestic risk factors for erosive tooth wear in the primary dentition. Int J Paediatr Dent 35(2):389–404. 10.1111/ipd.13250 39056584 10.1111/ipd.13250 PMC11788517

[9] Papagianni CE, van der Meulen MJ, Naeije M, Lobbezoo F (2013) Oral health-related quality of life in patients with tooth wear. J Oral Rehabil 40(3):185–190. 10.1111/joor.12025 23278167 10.1111/joor.12025

[10] Carvalho TS, Colon P, Ganss C, Huysmans MC, Lussi A, Schlueter N et al (2016) Consensus Report of the European Federation of Conservative Dentistry: Erosive tooth wear diagnosis and management. Swiss Dent J 126(4):342–346. 10.61872/sdj-2016-04-143 27142130 10.61872/sdj-2016-04-143

[11] Hughes JA, West NX, Addy M (2004) The protective effect of fluoride treatments against enamel erosion in vitro. J Oral Rehabil 31(4):357–363. 10.1046/j.1365-2842.2003.01240.x 15089942 10.1046/j.1365-2842.2003.01240.x

[12] Donovan T, Nguyen-Ngoc C, Abd Alraheam I, Irusa K (2021) Contemporary diagnosis and management of dental erosion. J Esthet Restor Dent 33(1):78–87. 10.1111/jerd.12706 33410255 10.1111/jerd.12706

[13] Buzalaf MAR, Hannas AR, Kato MT (2012) Saliva and dental erosion. J Appl Oral Sci 20(5):493–502. 10.1590/S167877572012000500001 23138733 10.1590/S1678-77572012000500001 PMC3881791

[14] Buzalaf MAR, Magalhães AC, Rios D, Wiegand A (2025) Prevention and Treatment of Dental Erosion: Beyond Fluorides. Monogr Oral Sci 33:216–227. 10.1159/000543569 40435950 10.1159/000543569

[15] Magalhães AC, Levy FM, Souza BM, Cardoso CAB, Cassiano LP, Pessan JP et al (2014) Inhibition of tooth erosion by milk containing different fluoride concentrations: an in vitro study. J Dent 42(4):498–502. 10.1016/j.jdent.2013.12.009 24373857 10.1016/j.jdent.2013.12.009

[16] Cassiano LP, Charone S, Souza JG, Leizico LC, Pessan JP, Magalhães AC et al (2016) Protective Effect of Whole and Fat-Free Fluoridated Milk, Applied before or after Acid Challenge, against Dental Erosion. Caries Res 50(2):111–116. 10.1159/000444024 26939048 10.1159/000444024

[17] Salas MMS, Nascimento GG, Huysmans MC, Demarco FF (2015) Estimated prevalence of erosive tooth wear in permanent teeth of children and adolescents: an epidemiological systematic review and meta-regression analysis. J Dent 43(1):42–50. 10.1016/j.jdent.2014.10.012 25446243 10.1016/j.jdent.2014.10.012

[18] Kensche A, Dürasch A, König B, Henle T, Hannig C, Hannig M (2019) Characterization of the in situ pellicle ultrastructure formed under the influence of bovine milk and milk protein isolates. Arch Oral Biol 104:133–140. 10.1016/j.archoralbio.2019.05.021 31202148 10.1016/j.archoralbio.2019.05.021

[19] Kensche A, Pohl C, Basche S, Dürasch A, Henle T, Hannig M, Hannig C (2025) Bovine milk and milk protein – promotor or inhibitor of bacterial biofilm formation at the tooth surface? BMC Oral Health 25:992. 10.1186/s12903-025-06432-1 40604707 10.1186/s12903-025-06432-1 PMC12219159

[20] Manton DJ, Cai F, Yuan Y, Walker GD, Cochrane NJ, Reynolds C et al (2010) Effect of casein phosphopeptide-amorphous calcium phosphate added to acidic beverages on enamel erosion in vitro. Aust Dent J 55(3):275–279. 10.1111/j.1834-7819.2010.01235.x 20887514 10.1111/j.1834-7819.2010.01234.x

[21] Schestakow A, Echterhoff B, Hannig M (2024) Erosion protective properties of the enamel pellicle in-situ. J Dent 147:105103. 10.1016/j.jdent.2024.105103 38815730 10.1016/j.jdent.2024.105103

[22] Shkembi B, Huppertz T (2023) Impact of dairy products and plant-based alternatives on dental health: food matrix effects. Nutrients 15(6):1469. 10.3390/nu15061469 36986199 10.3390/nu15061469 PMC10056336

[23] Ferrazzano GF, Cantile T, Quarto M, Ingenito A, Chianese L, Addeo F (2008) Protective effect of yogurt extract on dental enamel demineralization in vitro. Aust Dent J 53(4):314–319. 10.1111/j.1834-7819.2008.00072.x 19133946 10.1111/j.1834-7819.2008.00072.x

[24] Alcorta A, Porta A, Tárrega A, Alvarez MD, Vaquero MP (2021) Foods for plant-based diets: challenges and innovations. Foods 10(2):293. 10.3390/foods10020293 33535684 10.3390/foods10020293 PMC7912826

[25] Salehi G, Díaz E, Redondo R (2023) Forty-five years of research on vegetarianism and veganism: A systematic and comprehensive literature review of quantitative studies. Heliyon 9(5):e16091. 10.1016/j.heliyon.2023.e16091 37223710 10.1016/j.heliyon.2023.e16091 PMC10200863

[26] Asif N, Anwar O, Arif S, Anwar Z, Iahtisham-Ul-Haq, Ercisli S et al (2026) The rise of plant-based milk alternatives: exploring nutritional, health, and sustainability impacts. Food Chem X 34:103528. 10.1016/j.fochx.2026.103528 41630881 10.1016/j.fochx.2026.103528 PMC12861291

[27] Carey CN, Paquette M, Sahye-Pudaruth S, Dadvar A, Dinh D, Khodabandehlou K et al (2023) The environmental sustainability of plant-based dietary patterns: a scoping review. J Nutr 153(3):857–869. 10.1016/j.tjnut.2023.02.001 36809853 10.1016/j.tjnut.2023.02.001

[28] Ramsing R, Santo R, Kim BF, Altema-Johnson D, Wooden A, Chang KB et al (2023) Dairy and plant-based milks: implications for nutrition and planetary health. Curr Environ Health Rep 10:291–302. 10.1007/s40572-023-00400-z 37300651 10.1007/s40572-023-00400-z PMC10504201

[29] Turaga SS, Sukhabogi JR, Doshi D, Jummala S, Billa AL (2024) Comparing the effect of animal and plant-based yogurt extracts on enamel demineralization: an in vitro study. Minerva Dent Oral Sci 73(3):161–168. 10.23736/S2724-6329.23.04804-0 37381740 10.23736/S2724-6329.23.04804-0

[30] West NX, Hughes JA, Addy M (2000) Erosion of dentine and enamel in vitro by dietary acids: the effect of temperature, acid character, concentration and exposure time. J Oral Rehabil 27(10):875–880. 10.1046/j.1365-2842.2000.00583.x 11065022 10.1046/j.1365-2842.2000.00583.x

[31] Wiegand A, Attin T (2014) Randomised in situ trial on the effect of milk and CPP-ACP on dental erosion. J Dent 42(9):1210–1215. 10.1016/j.jdent.2014.07.009 25038509 10.1016/j.jdent.2014.07.009

[32] Hara AT, Zero DT (2014) Aetiology of dental erosion: patient-related factors. The potential of saliva in protecting against dental erosion. Monogr Oral Sci 25:197–205. 10.1159/000360372 24993267 10.1159/000360372

[33] Shellis RP, Featherstone JD, Lussi A (2014) Understanding the chemistry of dental erosion. Monogr Oral Sci 25:163–179. 10.1159/000359943 24993265 10.1159/000359943

[34] Esteves-Oliveira M, Witulski N, Hilgers RD, Apel C, Meyer-Lueckel H, Eduardo Cde P (2015) Combined Tin-Containing Fluoride Solution and CO_2_ Laser Treatment Reduces Enamel Erosion in vitro. Caries Res 49(6):565–574. 10.1159/000439316 26418736 10.1159/000439316

[35] Esteves-Oliveira M, Pasaporti C, Heussen N, Eduardo CP, Lampert F, Apel C (2011) Prevention of toothbrushing abrasion of acid-softened enamel by CO(2) laser irradiation. J Dent 39(9):604–611. 10.1016/j.jdent.2011.06.007 21741428 10.1016/j.jdent.2011.06.007

[36] Gerrard WA, Winter PJ (1986) Evaluation of toothpastes by their ability to assist rehardening of enamel in vitro. Caries Res 20(3):209–216. 10.1159/000260937 3456844 10.1159/000260937

[37] Esteves-Oliveira M, Wollgarten S, Liebegall S, Jansen P, Bilandzic M, Meyer-Lueckel H et al (2017) A New Laser-Processing Strategy for Improving Enamel Erosion Resistance. J Dent Res 96(10):1168–1175. 10.1177/0022034517718532 28665779 10.1177/0022034517718532

[38] Lee J, Park YS, Lee HJ, Koo YE (2022) Microwave-assisted digestion method using diluted nitric acid and hydrogen peroxide for the determination of major and minor elements in milk samples by ICP-OES and ICP-MS. Food Chem 373(Pt B):131483. 10.1016/j.foodchem.2021.131483 34782211 10.1016/j.foodchem.2021.131483

[39] Poitevin E, Nicolas M, Graveleau L, Richoz J, Andrey D, Monard F (2009) Improvement of AOAC Official Method 984.27 for the determination of nine nutritional elements in food products by Inductively coupled plasma-atomic emission spectroscopy after microwave digestion: single-laboratory validation and ring trial. J AOAC Int 92(5):1484–1518 19916387

[40] Aykut-Yetkiner A, Attin T, Wiegand A (2014) Prevention of dentine erosion by brushing with anti-erosive toothpastes. J Dent 42(7):856–861. 10.1016/j.jdent.2014.03.011 24704085 10.1016/j.jdent.2014.03.011

[41] Vertuan M, de Souza BM, Machado PF, Mosquim V, Magalhães AC (2020) The effect of commercial whitening toothpastes on erosive dentin wear in vitro. Arch Oral Biol 109:104580. 10.1016/j.archoralbio.2019.104580 31593890 10.1016/j.archoralbio.2019.104580

[42] Francese MM, Gonçalves IVB, Vertuan M, de Souza BM, Magalhães AC (2022) The protective effect of the experimental TiF_4_ and chitosan toothpaste on erosive tooth wear in vitro. Sci Rep 12(1):7088. 10.1038/s41598-022-11261-1 35490193 10.1038/s41598-022-11261-1 PMC9056515

[43] Francese MM, Urasaki BAN, de Barros MC, Ferrari CR, Grizzo LT, Magalhães AC (2024) Toothpaste containing TiF_4_ and chitosan against erosive tooth wear in situ. J Dent 145:104977. 10.1016/j.jdent.2024.104977 38582434 10.1016/j.jdent.2024.104977

[44] Ganss C, Marten J, Hara AT, Schlueter N (2016) Toothpastes and enamel erosion/abrasion - Impact of active ingredients and the particulate fraction. J Dent 54:62–67. 10.1016/j.jdent.2016.09.005 27650640 10.1016/j.jdent.2016.09.005

[45] Lucchese A, Bertacci A, Lo Giudice A, Polizzi E, Gherlone E, Manuelli M et al (2020) Stannous Fluoride Preventive Effect on Enamel Erosion: An In Vitro Study. J Clin Med 9(9):2755. 10.3390/jcm9092755 32858829 10.3390/jcm9092755 PMC7563875

[46] Fiorillo L, Cervino G, Herford AS, Laino L, Cicciù M (2020) Stannous Fluoride Effects on Enamel: A Systematic Review. Biomimetics (Basel) 5(3):41. 10.3390/biomimetics5030041 32878006 10.3390/biomimetics5030041 PMC7559150

[47] Nicholson JW (2025) Stannous Fluoride in Toothpastes: A Review of Its Clinical Effects and Likely Mechanisms of Action. J Funct Biomater 16(3):73. 10.3390/jfb16030073 40137352 10.3390/jfb16030073 PMC11942899

[48] Babcock FD, King JC, Jordan TH The reaction of stannous fluoride and hydroxyapatite. J Dent Res 1978 Sep-Oct ; 57(9–10): 933–938. 10.1177/00220345780570092301 10.1177/00220345780570092301 281374

[49] Boeck T, Sahin AW, Zannini E, Arendt EK (2021) Nutritional properties and health aspects of pulses and their use in plant-based yogurt alternatives. Compr Rev Food Sci Food Saf 20:1–23. 10.1111/1541-4337.12778 10.1111/1541-4337.12778 34125502

[50] Santos EJLD, Meira IA, Sousa ET, Amaechi BT, Sampaio FC, Oliveira AFB (2019) Erosive potential of soy-based beverages on dental enamel. Acta Odontol Scand 77(5):340–346. 10.1080/00016357.2019.1570330 30741104 10.1080/00016357.2019.1570330

[51] Romão DA, de O Santos INA, Paes LR, de C Santos MR, Caju GBL, dos Santos VE Júnior et al (2025) Analysis of Chemical Properties of Fermented Milk Beverages Containing Probiotics and Influence on Enamel Demineralization: An in Vitro Study. Pesqui Bras Odontopediatria Clín Integr 25:e230166. 10.1590/pboci.2025.002

[52] Lussi A, Carvalho TS (2014) Erosive tooth wear: a multifactorial condition of growing concern and increasing knowledge. Monogr Oral Sci 25:1–15. 10.1159/000360380 24993253 10.1159/000360380

[53] Basu SN, Kumar P, Gawali RA, Urs AB (2025) Protective Efficacy of Yogurt, Milk, and Fluoridated Tooth Creme Against Acidic Beverages on Human Teeth: Stereomicroscopic and Ultrastructural Analyses. Microsc Res Tech 88(6):1835–1847. 10.1002/jemt.24819 39957588 10.1002/jemt.24819

[54] Larnani S, Song Y, Kim S, Park YS (2025) Examining enamel-surface demineralization upon exposure to acidic solutions and the remineralization potential of milk and artificial saliva. Odontology 113(1):201–212. 10.1007/s10266-024-00960-y 38904919 10.1007/s10266-024-00960-y

